# Noble Metal Nanoparticle Biosensors: From Fundamental Studies toward Point-of-Care Diagnostics

**DOI:** 10.1021/acs.accounts.1c00598

**Published:** 2022-02-09

**Authors:** Hongya Geng, Simon Vilms Pedersen, Yun Ma, Tabasom Haghighi, Hongliang Dai, Philip D. Howes, Molly M. Stevens

**Affiliations:** Department of Materials, Imperial College London, London SW7 2AZ, U.K.; Department of Medical Biochemistry and Biophysics, Karolinska Institute, Stockholm 171 77, Sweden; Department of Materials, Imperial College London, London SW7 2AZ, U.K; Department of Materials, Imperial College London, London SW7 2AZ, U.K; Department of Materials, Imperial College London, London SW7 2AZ, U.K; School of Environmental and Chemical Engineering, Jiangsu University of Science and Technology, Zhenjiang 212003, China; Division of Mechanical Engineering and Design, School of Engineering, London South Bank University, London SE1 0AA, U.K

## Abstract

Noble metal nanoparticles (NMNPs) have become firmly established as effective agents to detect various biomolecules with extremely high sensitivity. This ability stems from the collective oscillation of free electrons and extremely large electric field enhancement under exposure to light, leading to various light–matter interactions such as localized surface plasmon resonance (LSPR) and surface-enhanced Raman scattering. A remarkable feature of NMNPs is their customizability by mechanisms such as particle etching, growth, and aggregation/dispersion, yielding distinct color changes and excellent opportunities for colorimetric biosensing in user-friendly assays and devices. They are readily functionalized with a large variety of capping agents and biomolecules, with resultant bioconjugates often possessing excellent biocompatibility, which can be used to quantitatively detect analytes from physiological fluids. Furthermore, they can possess excellent catalytic properties that can achieve significant signal amplification through mechanisms such as the catalytic transformation of colorless substrates to colored reporters. The various excellent attributes of NMNP biosensors have put them in the spotlight for developing high-performance in vitro diagnostic (IVD) devices that are particularly well-suited to mitigate the societal threat that infectious diseases pose. This threat continues to dominate the global health care landscape, claiming millions of lives annually. NMNP IVDs possess the potential to sensitively detect infections even at very early stages with affordable and field-deployable devices, which will be key to strengthening infectious disease management. This has been the major focal point of current research, with a view to new avenues for early multiplexed detection of infectious diseases with portable devices such as smartphones, especially in resource-limited settings.

In this Account, we provide an overview of our original inspiration and efforts in NMNP-based assay development, together with some more sophisticated IVD assays by ourselves and many others. Our work in the area has led to our recent efforts in developing IVDs for high-profile infectious diseases, including Ebola and HIV. We emphasize that integration with digital platforms represents an opportunity to establish and efficiently manage widespread testing, tracking, epidemiological intelligence, and data sharing backed by community participation. We highlight how digital technologies can address the limitations of conventional diagnostic technologies at the point of care (POC) and how they may be used to abate and contain the spread of infectious diseases. Finally, we focus on more recent integrations of noble metal nanoparticles with Raman spectroscopy for accurate, noninvasive POC diagnostics with improved sensitivity and specificity.

**Figure:**
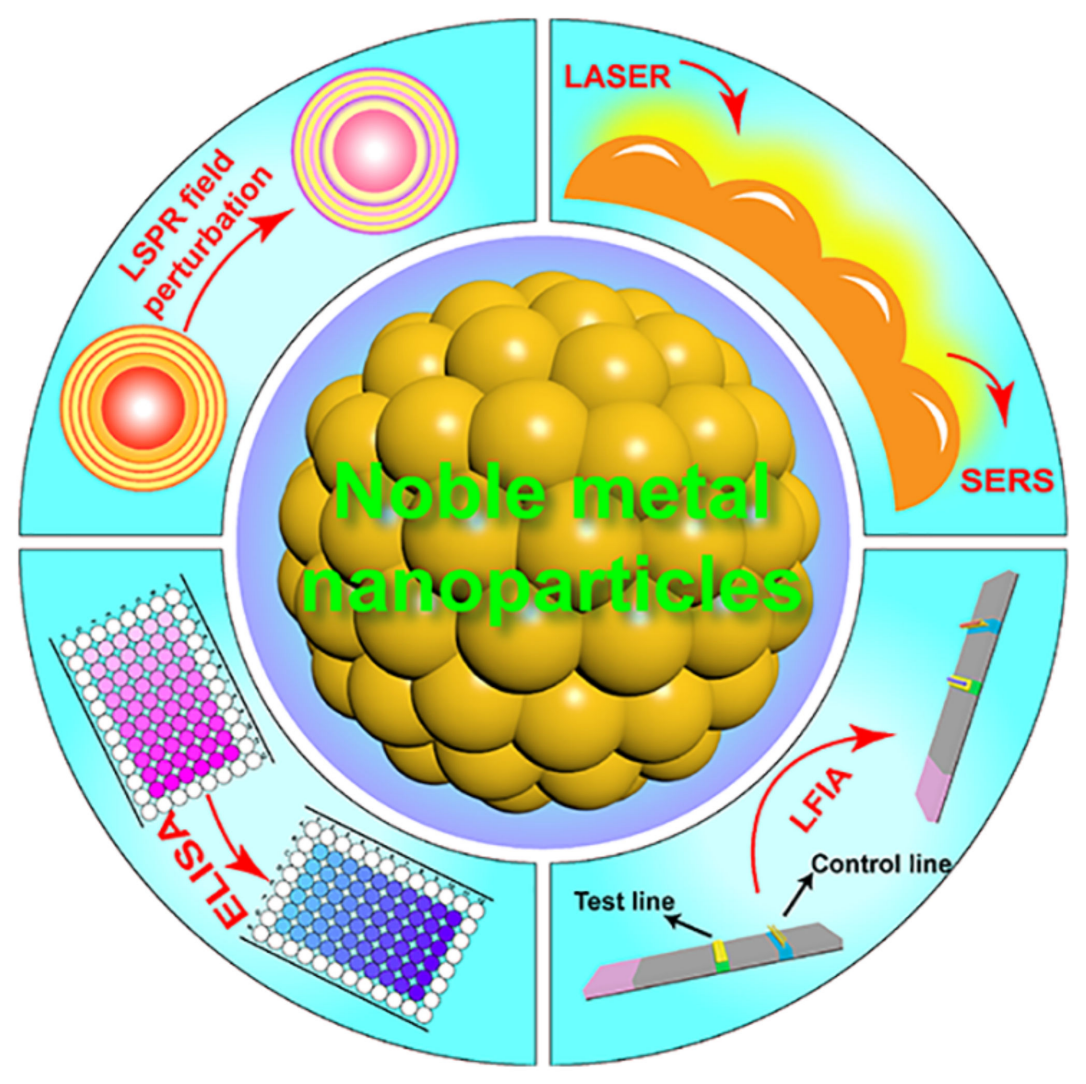

## 1 Introduction

Noble metal nanoparticles (NMNPs) have attracted significant attention for early disease diagnosis through biomarker detection with extremely high sensitivity.^5^ In biosensing applications, they are frequently used due to their unique surface plasmon resonance (SPR). SPR is characterized by local charge oscillation in resonance with incident light when the size of particles is smaller than the light wavelength. The local charge oscillation can confine light at nanoscale dimensions, by which scattering and absorption can be significantly enhanced.^6^ Importantly, these NPs exhibit distinct and sensitive changes in optical properties as they aggregate/disaggregate, change shape by etching or growth, or experience other changes in their local environment. NMNPs can be functionalized with a wide range of capping agents, which provides great versatility in many molecular detection scenarios. Further, some NMNPs exhibit natural enzyme-like catalytic properties, which allows a single NP to generate many reporters, for example producing colored products from colorless precursors, allowing pronounced signal amplification.^7^

Over recent decades, the frequency and amplitude of infectious disease outbreaks have increased.^8^ The death toll of the ongoing severe acute respiratory syndrome coronavirus (COVID-19) pandemic surpassed 4 million during the writing of this Account^9^ and represents the third coronavirus outbreak in less than 20 years after MERS in 2012 and SARS in 2003.^10,11^ The Ebola virus outbreak in West Africa in 2013 claimed over 11000 lives,^12^ and the human immunodeficiency virus (HIV) and severe Zika virus outbreaks have sparked grave concerns about global health.^13,14^ Since infectious diseases can be rapidly transmitted among humans by live pathogens such as airborne or waterborne viruses, parasites, and bacteria, early stage field-deployable in vitro diagnostics (IVDs) play a pivotal role in cutting off the transmission routes, helping to protect susceptible people and monitor population recovery.

**Figure 1:**
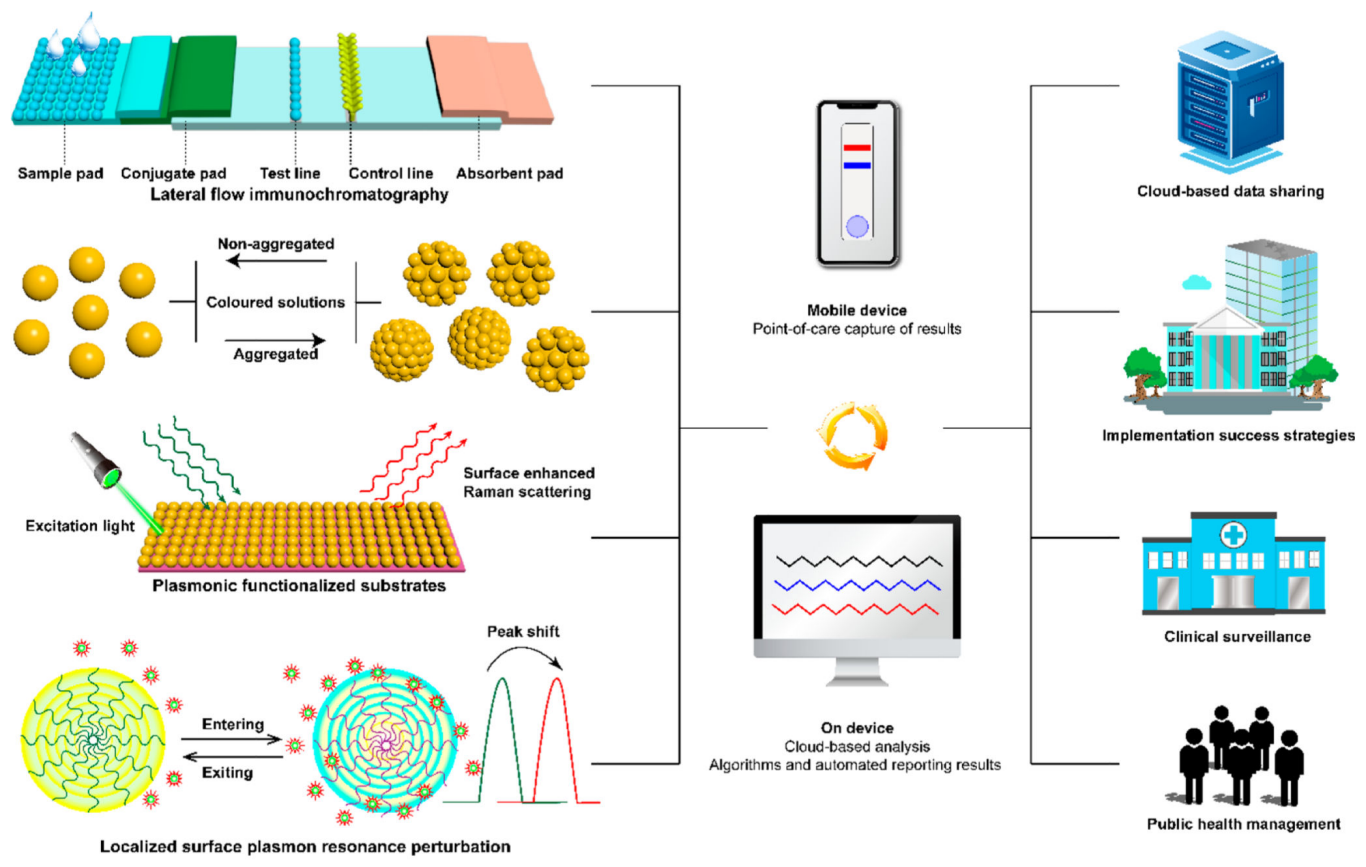
Deploying noble metal nanoparticles with integrated digital technologies drives early infectious disease detection and diagnosis.

Despite the high sensitivity and specificity of polymerase chain reaction (PCR) and enzyme-linked immunosorbent assay (ELISA) for detection of infections (e.g., through genetic materials, pathogen-related proteins, and human host generated pathogen-specific antibodies), they require skilled professionals, sophisticated infrastructure, and costly reagents, impeding their translation into clinical environments, and more so in low-resource settings.^15^ In contrast, lateral flow assays (LFAs) detect biomolecules on a disposable nitrocellulose strip, where a colorimetric response generally allows equipment-free rapid readout, often within 5 min (Figure 1). These advantages have popularized LFAs for point-of-care (POC) infectious disease diagnosis.

This Account provides an overview of our work and experience in developing NMNP biosensors and also discusses some of the excellent work from other groups. We emphasize advanced diagnostic strategies based on aggregation/disaggregation, anisotropic growth, liposome-mediated aggregation, and signal amplification of NMNPs that arise from their plasmonic and catalytic activities. Further, we provide suggestions and perspectives on integrating NMNPs with portable devices, such as smartphones and LFAs, to improve POC testing. The potential in exploiting surface-enhanced Raman scattering using NMNPs for diagnostics is also proposed.

## 2 Fundamental Mechanism of Noble Metal Nanoparticles as Biosensors

This Account covers two main mechanisms whereby NMNPs can detect analytes (Figure 1): (1) surface etching, and anisotropic growth-induced visible color change in the presence of analytes, arising due to position or shape changes, or both, of the localized surface plasmon resonance (LSPR) peak(s) in the visible range, which occurs because the plasmonic character of nanostructures is strongly dependent on particle shape and size, interparticle distance, environment, etc.;^16^ (2) aggregation/disaggregation and shifting of the LSPR peak due to non-destructive changes in the local environment around particles.^17^ We also briefly discuss how the measurement of Raman intensities, which can be modulated by binding or removing Raman-active probes from NMNPs, has great potential for future use in biosensing. Here, small variations in either the number density of molecules being probed or the arrangement of metal atoms within the nanostructures have a significant effect on Raman intensities.^18^ In principle, the sensitivity of such methods is increased if a single analyte molecule leads to a cascade of chemical events amplifying response signals due to the NMNP-catalyzed reactions.^19^ A fourth mechanism of interest is noble metal-enhanced fluorescence and quenching, where fluorescence intensity can be modulated by the distance between the fluorescent reporter and the noble metal surface through the interaction of the excited state with the plasmonic field.^20^ We do not cover this mechanism in this Account, but refer interested readers to several recent examples instead.^21–23^

**Figure 2:**
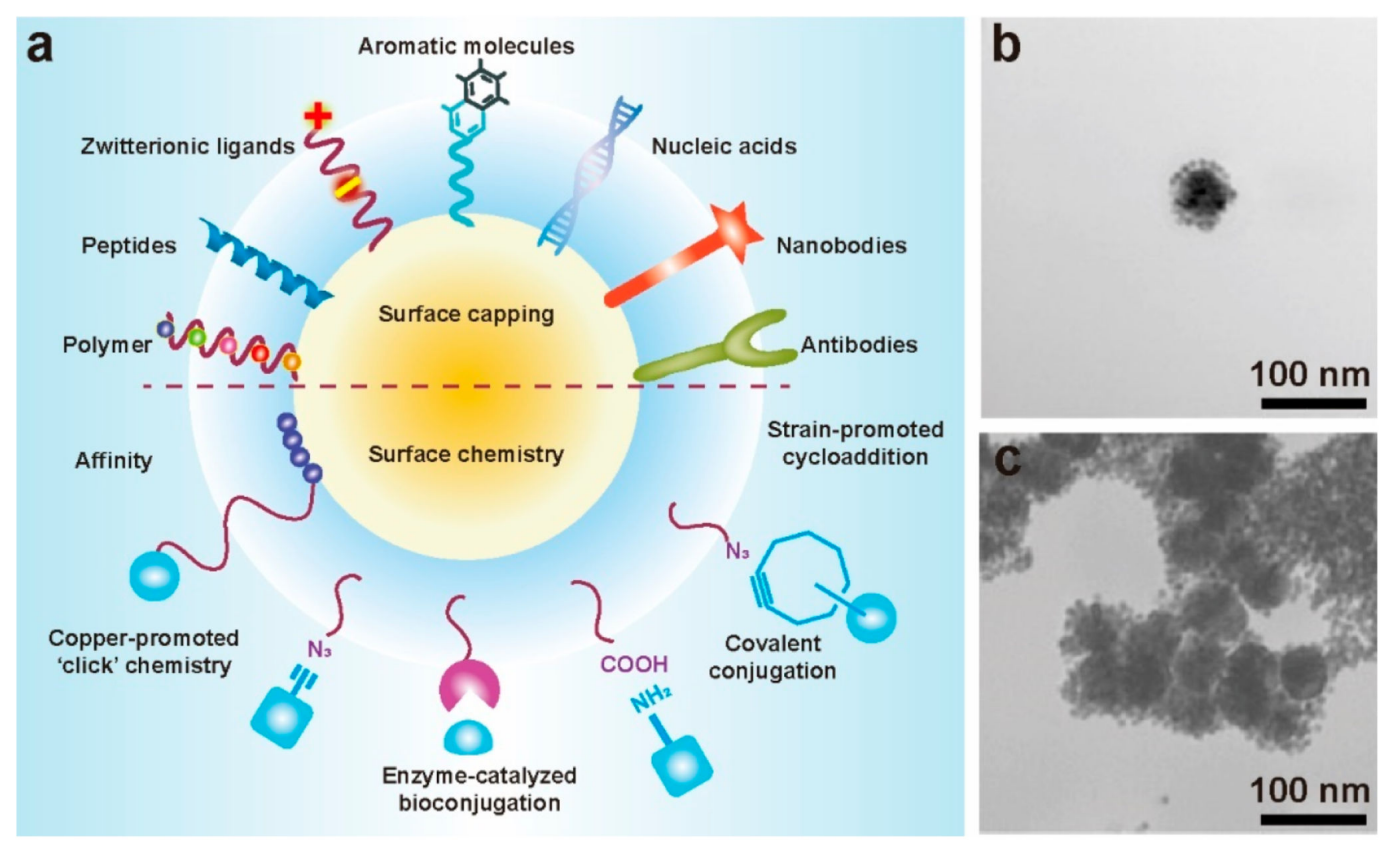
(a) Bioconjugation of ligands providing colloidal stability and functional groups targeting biomolecules. Adapted with permission from ref 5. Copyright 2014 American Association for the Advancement of Science. (b, c) Typical TEM images showing the self-assembly of peptide coated AuNPs. Reproduced with permission from ref 24. Copyright 2004 WILEY-VCH Verlag GmbH & Co. KGaA, Weinheim.

The surface composition of nanoparticles is key in controlling their behavior in all analyte detection scenarios, most often through bioconjugation of recognition elements such as antibodies, nucleic acids, aptamers, proteins, and peptides (Figure 2a). These often confer the extra benefit of enhancing colloidal stability, especially when paired with advanced ligand chemistries, for example, employing zwitterionic or polymeric capping layers.^5^

Our initial development of NMNP biosensors grew out of historical interest in studying functional peptides for biomedical applications.^25^ Artificial bioactive peptides can exhibit versatile and programmable protein-like functionalities while possessing a high degree of flexibility in molecular design and manufacturing.^26,27^ The screening of peptide libraries can facilitate the discovery of effective binders against a large variety of analytes with high specificity, including cells, RNAs, DNAs, proteins, and other peptides.^28^ Ran et al. enabled the simultaneous detection of multiple inflammatory biomarkers over a broad range of concentrations, from pg/mL to μg/mL, using peptide-mediated aggregation of AuNPs.^29^ Three phosphorylated peptides possessing different responses to alkaline phosphatase (ALP) were used. These influenced the degree of AuNP aggregation differently, due to the difference of the ALP hydrolysis efficiency and the electrostatic and spatial interactions between the peptides and the AuNPs. More sensitive peptides could induce aggregation of AuNPs in solutions with lower ALP, and vice versa. Therefore, biomarkers that present at a broad range of concentrations could be detected in the presence of a suitable peptide and ALP, linked to the detection antibody of the corresponding biomarker, by monitoring the aggregation of AuNPs. Additionally, we established an early platform for biomolecular recognition based on the functionality of coiled-coil peptides conjugated to gold NPs (AuNPs) (Figure 2b,c).^24^ Either an acidic leucine zipper-like peptide or a basic leucine zipper-like peptide were separately conjugated onto AuNPs via terminal cysteine residues. Hydrophobic packing between these peptides drove the formation of a left-handed coiled-coil structure between the leucine zipper-like domains. The oppositely charged lysine–glutamate ion pairs on the coiled-coil structure were able to dynamically modulate the stability of the coiled coils through electrostatic interactions in response to pH. The approach of dynamic control of the assembly of AuNPs via various mechanisms is of great interest for biosensing. For example, from this fundamental study we developed a novel strategy for real-time monitoring of protease activity with a single population of protease-responsive AuNPs.^30^ A tripeptide was designed and functionalized onto AuNPs. The peptide (Fmoc-Gly↓Phe-Cys-NH_2_) contained a cysteine-containing thiol group to anchor it to the AuNPs surface, a scissible bond at the amine side of Gly-Phe served as a protease-cleavable unit, and N-(fluorenyl-9-methoxycarbonyl) at the outer end of the peptide to facilitate AuNP aggregation through π-stacking interactions. Upon enzymatic hydrolysis by thermolysin, removal of hydrophobic interactions between Fmoc groups allowed disaggregation of the AuNPs and a visible solution color change from blue to red.

These early studies in NMNP bioconjugate design and fabrication further enabled the development of a colorimetric kinase detection assay.^31^ This system consisted of two populations of AuNPs: one coated with protein kinase substrate peptide, and the other with complementary antiphosphotyrosine antibodies. The addition of kinase caused enzymatic phosphorylation of the protein kinase peptide-capped AuNPs, which facilitated interparticle assembly as the antibodies bound to the modified peptide with high specificity.

## 3 Advanced Strategies that Govern the Detection of Infectious Diseases

### 3.1 Anisotropic Growth and Etching of the NMNPs

Since our early assays of peptide-mediated AuNP assembly, we have started exploiting shifts in plasmonic peaks (and therefore colorimetric response) resulting from structural changes of NMNPs (size, shape, interparticle spacing, and particle composition), giving rise to extremely sensitive diagnostics assays.

A fundamental mechanism utilized in NMNP biosensors is the colorimetric response arising from a shift of plasmonic peaks. This was harnessed in an early assay of ours, where we lowered the limit of detection (LOD) of a cancer biomarker (prostate specific antigen, PSA) via the controlled overgrowth of Ag on Au nanostars.^32^ In other work we developed a plasmonic ELISA, where enzyme-mediated growth of AuNPs allowed extremely sensitive detection of HIV-1 capsid antigen p24.^33^ Similar to conventional ELISA, p24 was first captured on the plates using capture antibodies (human monoclonal anti-p24); then primary and secondary antibodies were added to link p24 to streptavidin–catalase conjugates selectively. Catalase bound in the ELISA was able to modulate the amount of H_2_O_2_ per well affecting the AuNP growth.^34^ Naked-eye detection of HIV-1 capsid antigen p24 (from 20 HIV-infected patients) as low as 1 × 10^−18^ g mL^−1^ was realized.

**Figure 3:**
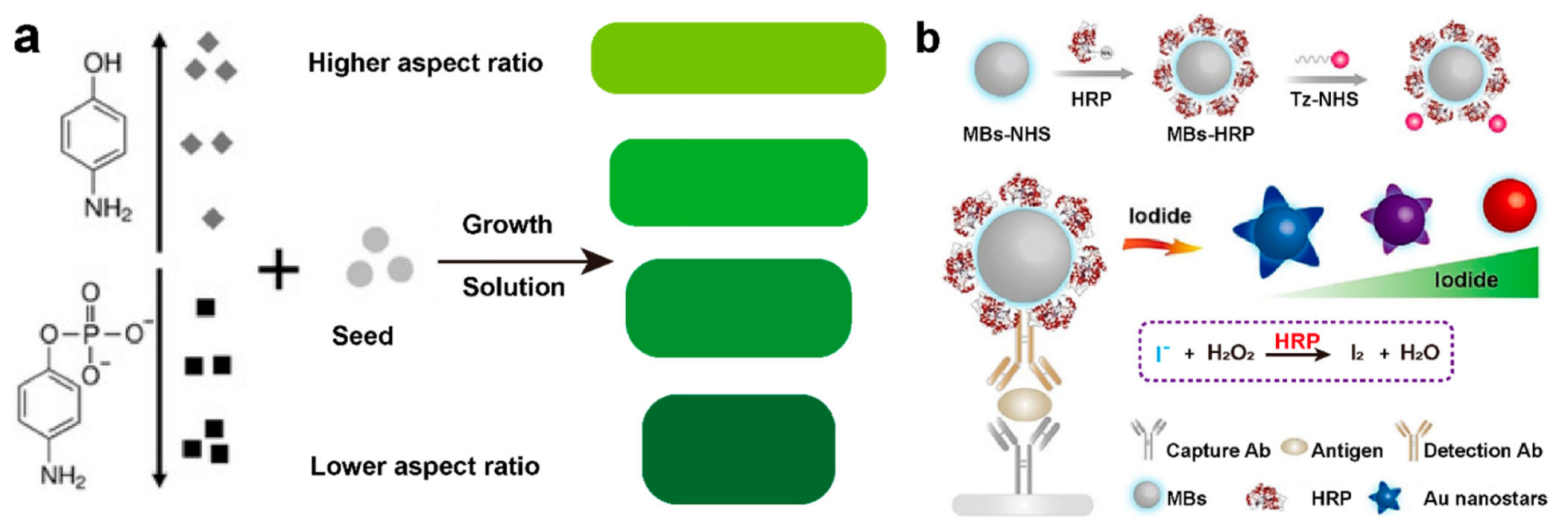
(a) Schematic illustration of the concentration-dependent effects of AP and APP on the aspect ratio of AuNRs. Adapted with permission from ref 35. Copyright 2017 WILEY-VCH Verlag GmbH & Co. KGaA, Weinheim. (b) Illustration of a sandwich immunoassay based on the surface etching of the Au nanostars. Reproduced with permission from ref 37. Copyright 2021 WILEY-VCH Verlag GmbH & Co. KGaA, Weinheim.

We have also taken advantage of the anisotropic growth of Au nanorods (AuNRs) to detect analytes.^35^ The LSPR modes along the longitudinal and transverse axes of these nanorods cover the visible and near-infrared regions, allowing ready and broad spectral adjustment through aspect ratio tuning (Figure 3a).^36^ Our results showed that high concentrations of reducing molecules, such as 4-aminophenol (AP), resulted in a higher number of seeds, promoting the extent of AuNRs during the growth process. This was reflected by a red shift of the produced nanorods. Capping molecules, such as 4-amino-phenyl phosphate (APP), inhibited the anisotropic growth of AuNRs, yielding a blue shift. We exploited this concept to detect PSA in a sandwich ELISA, where a higher concentration of PSA increased the conversion of APP to AP, leading to the growth of AuNRs with a higher aspect ratio. A red shift in longitudinal LSPR during the growth of AuNRs yielded an LOD of PSA at 0.16 ng mL^−1^.

In another study, we demonstrated an enzyme biosensor by reshaping Au nanostars using iodide etching (Figure 3b).^37^ The higher binding energy of I^–^ to Au surfaces compared to other halide ions enabled iodide-specific surface etching due to an electrochemical reaction.^38^ Gold nanostars could be variably etched as a function of iodide concentration, from just slight rounding of nanostar points (low iodide concentration) through to heavily etched spherical structures (high iodide concentration). In the presence of H_2_O_2_, horseradish peroxidase (HRP) catalyzed the oxidation of iodide into iodine, which cannot etch Au nanostars. A versatile plasmonic ELISA was developed based on this HRP-modulated etching, to detect human IgG as a protein model. Goat anti-human IgG (capture antibody), and a rabbit anti-human IgG (detection antibody) formed the sandwich pair for detection of human IgG. The detection antibody and HRP were cofunctionalized with magnetic beads (MBs, ~26530 HRP per MB) to catalyze iodide consumption and increase the HRP labelling of each target human IgG. As human IgG increased, more antibody functionalized HRP-MBs were captured by the plate to consume iodide. By monitoring changes in transverse and longitudinal bands at 550 and 780 nm, we achieved a LOD of 0.2 ng mL^−1^.

Similarly, Suea-Ngam et al. detected methicillin-resistant *Staphylococcus aureus* (MRSA) based on selective etching of Ag nanoplates.^39^ Loop-mediated isothermal amplification (LAMP) was used to amplify the target MRSA gene *(mecA)* from a test sample on a paper-based analytical device. The long LAMP DNA amplicons bound to Ag nanoplates on the strip and inhibited etching by NaBr. In the absence of the target gene, the silver nanoplates were not protected and were rapidly etched by the salt, leading to a dramatic shift in the plasmonic peak and color change from blue to yellow-orange. Gu et al. reported an assay for microRNA-141 in horse serum based on the SPR change of silver-coated gold nanorods (Au@Ag NRs).^40^ In response to microRNA-141, hydroxyl radicals generated from H_2_O_2_ due to the catalyzed hairpin assembly and hybridization chain reactions etched the Ag shell of the NRs. As low as 1.0 × 10^−14^ M microRNA could be quantified this way.

### 3.2 Aggregation Assays

Reversible biological assembly (aggregation) of NMNPs using highly specific biomolecular recognition systems yields distinct shifts in LSPR, valuable for sensitive biosensing.^24,41^ As a means of targeting specific analytes, the interactions between NMNPs and analytes can be driven by various mechanisms, including (1) highly specific antigen–antibody interactions,^4^ (2) enzyme substrates containing proteins or peptides that can be removed by protease cleavage,^42^ (3) charge–charge and hydrogen bonding interactions between nanoparticles and polymers,^43^ (4) various types of aptamers that target ions, biotoxins, and biomarkers,^44^ and (5) primers that can guide the amplification of DNA when polymerase and nucleotides are present^45^.

Using highly selective antigen–antibody interactions to trigger aggregation of AuNPs and detection of antibodies and antigens is of particular interest for infectious disease management. Antibody response can be used to detect later-stage active infections, a past infection, ongoing immunity status, or response to vaccination. Accordingly, we developed a rapid and sensitive assay for IgG antibodies employing aggregation of peptide epitope-capped AuNPs.^46^ The bivalent action of the tested monoclonal IgG antibodies led to direct cross-linking of epitope-tagged AuNPs and a subsequent red-shift that was proportional to the antibody concentration. For example, additions of 1–50 nM of an anti- *Haemophilus influenzae* mAb into AuNP dispersions caused a drop in plasmon peak intensity and an immediate red shift, while 100–300 nM mAbs resulted in extensive red shift and broadening of the plasmon peak. This assay enabled the detection of antibody levels of in low nanomolar concentrations which is relevant for the detection of disease specific antibodies typically circulating at serum levels of 0.01–10000 *μg* per mL.

A conceptually related mechanism was reported recently in application to the diagnosis of severe acute respiratory syndrome-coronavirus-2 (SARS-CoV-2) virus. Prikshit et al. designed AuNPs capped with thiol-modified antisense oligonucleotides that aggregate in the presence of the N-gene of target RNA of SARS-CoV-2.^47^ The selective agglomeration of AuNPs occurred upon the addition of thermostable RNaseH that specifically cleaves the phosphodiester bonds of the N-gene strand. The visible precipitate in solution, and the absorbance at 660 nm of AuNPs treated with SARS-CoV-2, gave a LOD down to 0.18 ng *μ*L^−1^. Many other bioconjugate formats can be explored that have similar effects. For example, Zheng et al.^48^ monitored the color change of aggregating AuNPs to indicate different concentrations of *Escherichia coli*, where aggregation was triggered by mixing AuNPs with crosslinking agents (phenolic hydroxyl moieties in tyramine) in the presence of capture antibody-capped magnetic NPs and detection antibody-modified polystyrene microspheres. Here the color change could be monitored in a microfluidic chamber with catalase and *Escherichia coli* cells at concentrations as low as 50 CFU/mL within 1 h.

**Figure 4:**
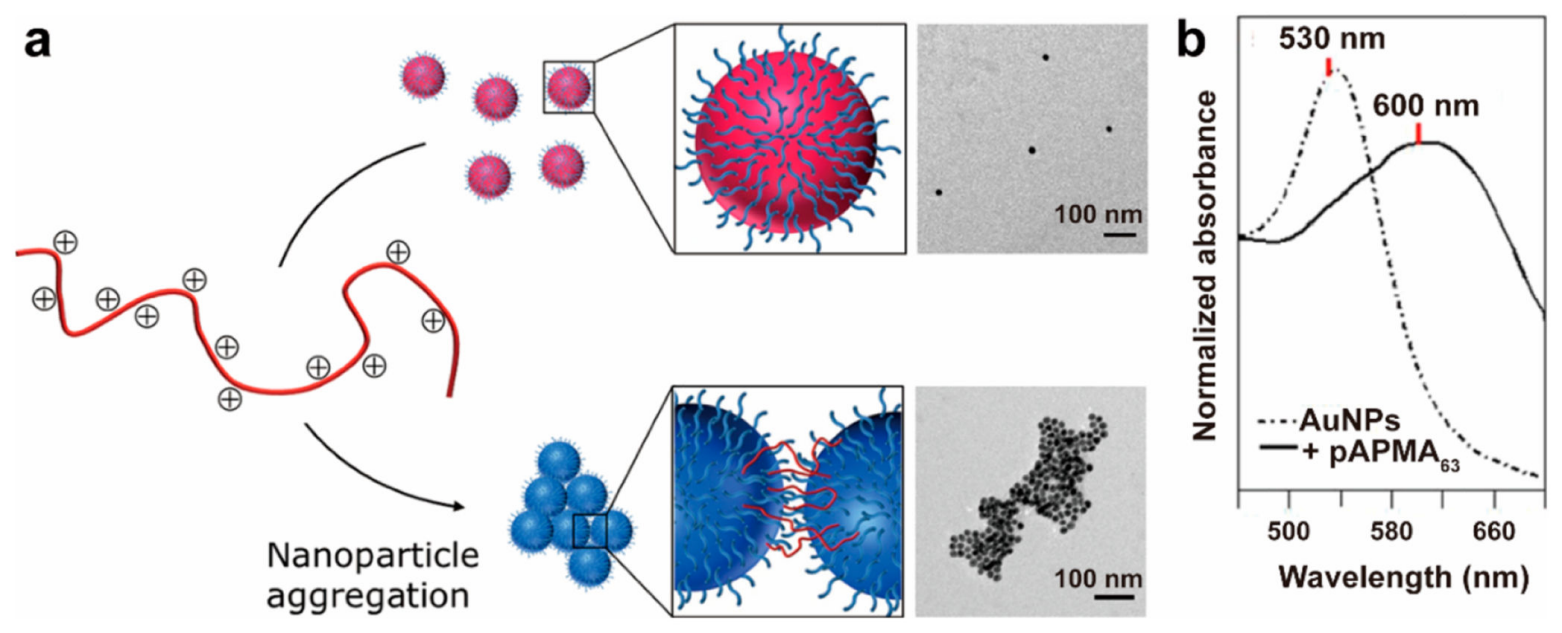
(a) Schematic illustration of an assay design that utilizes polymerization-based signal amplification. (b) Absorbance spectra of dispersed AuNPsand aggregated AuNPs due to the presence of pAPMA. Reproduced with permission from ref 49. Copyright 2014 American Chemical Society.

Building on previous aggregation-based assays, we designed a polymerization-based platform aimed toward enzyme (e.g., HRP and catalase) and ion (e.g., iron and copper) detection, as these can generate radicals to form polymers that entangle AuNPs into aggregates (Figure 4a).^49^ This polymer-mediated assay offers a promising signal amplification strategy. A cascade initiated in the presence of a very low concentration of free radicals can trigger AuNP aggregation by formation of large polymer chains, resulting in a visible color change (Figure 4b). For example, 1 *μ*g mL^−1^ of HRP could be detected as it triggers the polymerization of 3-aminopropyl methacrylamide, and we achieved a LOD of catalase down to 0.7 ng mL^–1^ using an inverse assay format.

### 3.3 Liposome-Triggered Aggregation Assay

Although the colorimetric response of NMNPs has been demonstrated in many systems, a compromise is often made between nonspecific interaction with the substrates and particle stability. The liposome provides a multifunctional platform that can be activated to release cargos such as DNA, polypeptides, and proteins to guide the assembly/disassembly process of NPs.

**Figure 5:**
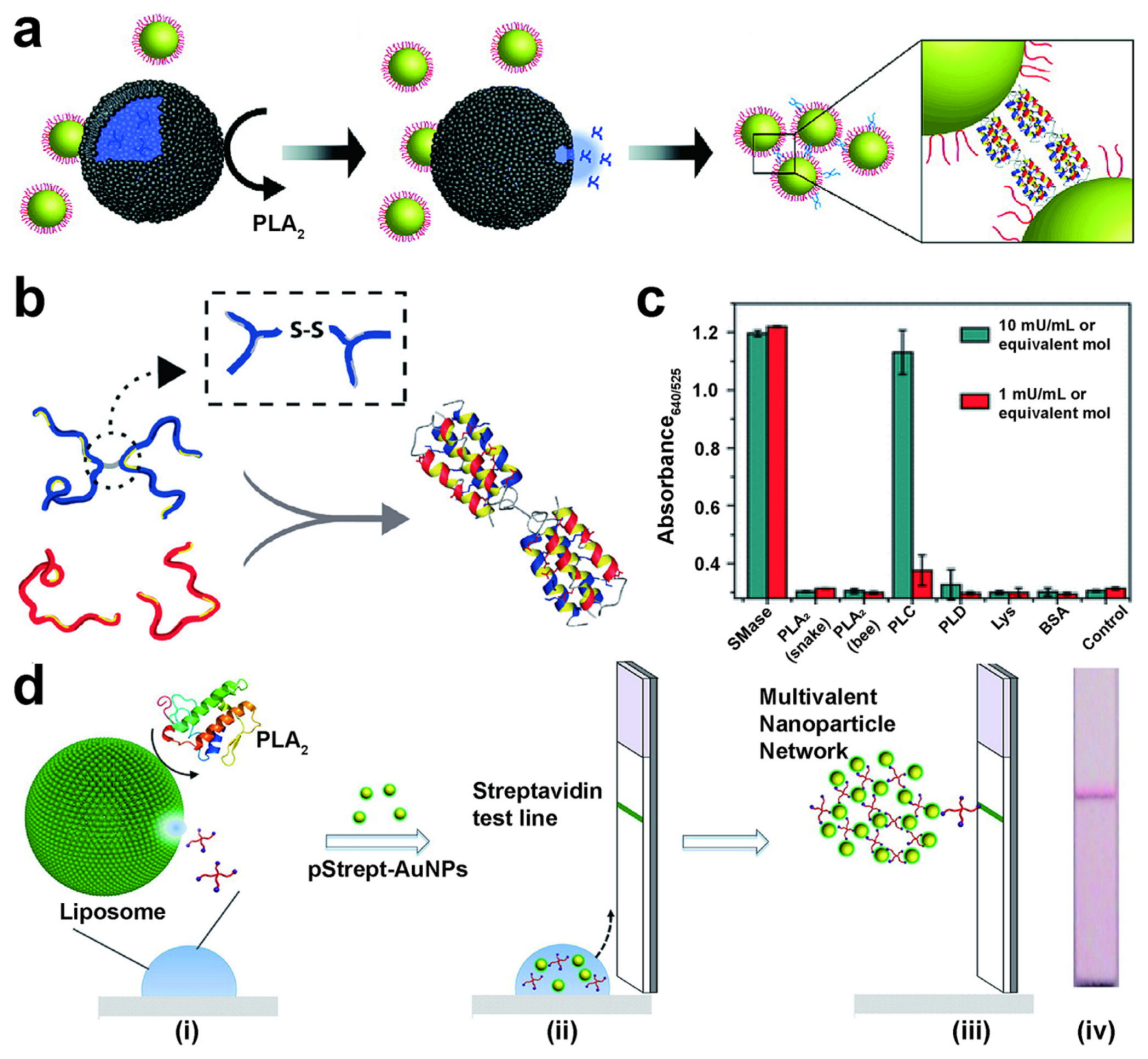
(a) Mechanism of PLA_2_ detection based on the release of JR2KC_2_ from a liposome. Reproduced with permission from ref51. Copyright 2011 American Chemical Society. (b) Formation of AuNP assemblies based on heteroassociation between JR2KC and glutamate-rich helix–loop–helix polypeptide. Reproduced with permission from ref52. Copyright 2011 American Chemical Society. (c) Exploring specificity for an SMase liposome-based assay in reaction buffer containing SMase, or denatured SMase, Triton X100, PLA_2_, phospholipase D (PLD), phospholipase C (PLC), lysozyme (Lys), and bovine serum albumin (BSA). Reproduced with permission from ref53. Copyright 2018 American Chemical Society. (d) LFA device schematic based on a modified liposome-triggered assay. Reproduced with permission from ref54. Copyright 2015 American Chemical Society.

Degradation of liposomes can be triggered by phospholipases, enzymes involved in various physiological processes such as inflammatory responses and intercellular signaling. Phospholipases act preferentially on lipid bilayers and micelles rather than free lipid monomers. One class of phospholipases, phospholipase A_2_ (PLA_2_), have been extensively demonstrated as biomarkers of many infectious conditions such as bacterial sepsis,^50^ which is why we developed a liposome-triggered NMNP aggregation assay for PLA_2_.^51^ Our system comprised polypeptide-functionalized AuNPs (JR2EC) and complementary polypeptide (JR2KC_2_). The 42-residue JR2EC could form bridges with nanomolar amounts of JR2KC_2_, causing rapid assembly of AuNPs (Figure 5a). When exposed to PLA_2_, the liposomes were degraded, releasing the pre-encapsulated JR2KC_2_, enabling aggregation of AuNPs and real-time monitoring of the significant red shift. In a rapid testing format, monitoring aggregation of AuNPs allowed us to detect PLA_2_ at concentrations down to 700 pM. We then undertook detailed characterization of this highly controllable assembly of AuNPs using UV-vis spectroscopy and small-angle X-ray scattering, which confirmed that this system forms regularly ordered aggregates with well-defined particle spacing that corresponds to the size of the folded four-helix bundles.^52^

In a related study, we demonstrated cysteine release from sphingomyelinase-based liposomes, driving AuNP aggregation to detect the sphingomyelinase enzyme (SMase), a biomarker of several infectious diseases including HIV.^53^ Here, a red AuNP dispersion was first added to a solution of cysteine preloaded liposomes. Cysteine is a small zwitterionic molecule with a thiol moiety that strongly binds gold.^55^ In the presence of SMase, cysteine was released as the liposome was enzymatically hydrolyzed into ceramide and phosphocholine. The released cysteine caused the aggregation of AuNPs through zwitterionic electrostatic interactions and hydrogen bonding between cysteines as they bound onto AuNPs. The color change from red to blue after aggregation was evident by peak broadening. Accordingly, we used the ratio between absorbance at 525 and that at 640 nm to determine the concentration of SMase. The LOD determined using the absorbance ratio was 0.02 mU mL^−1^ (1.4 pM enzyme concentration). Compared to other common phospholipases, the liposome-triggered aggregation exhibited high selectivity (Figure 5c).

Further developing the liposome-based aggregation assay concept, we devised a rapid lateral flow assay for PLA_2_ detection in serum using adhesion of polystreptavidin-coated AuNPs (Figure 5d).^54^ Here, PLA_2_ triggered the release of a biotinylated four-armed poly(ethylene glycol) (PEG) from the liposomes due to the phospholipid cleavage. Our design demonstrated detection of 1–10 nM of PLA_2_ in sera samples from acute pancreatitis patients, which was lower than a commercial kit (50 nM). This design also offers high flexibility, as changing the liposome components could tune it toward various natural enzyme activities. For example, we utilized a secretory phospholipase A2 group IIA (sPLA_2_-IIA)-based liposome substrate to release the four-armed PEG in LFA devices for POC measurement of sPLA_2_-IIA, which is associated with rheumatoid arthritis.^56^ The concentration of sPLA_2_-IIA in healthy serum measured using our LFA was 21.9 ± 23.9 ng mL^−1^, in line with ELISA analysis. Our simple LFA-based method should be an attractive alternative for rapid testing outside clinical settings, given that there is no need for laboratory facilities.

### 3.4 Signal Amplification Based on Catalytic NMNPs

**Figure 6:**
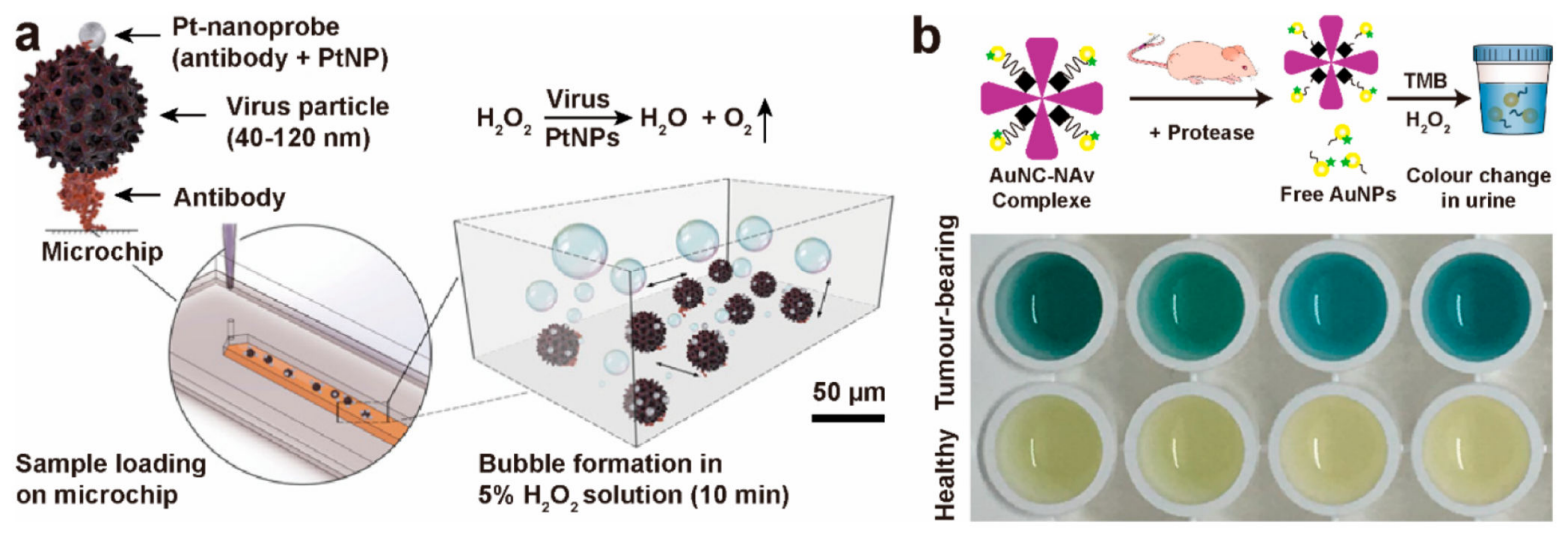
(a) Detection of virus particles on a microchip by bubble formation in the presence of PtNPs and H_2_O_2_. Reproduced from ref 61. Copyright 2020 The Authors. (b) Design of a renally clearable sensing system based on nanocatalyst signal amplification. Photograph shows the colorimetric assay of urine from tumor-bearing (top) and healthy mice (bottom). Reproduced from ref 2. Copyright 2019 The Authors.

All of the assays discussed so far have utilized the plasmonic properties of NMNPs to generate colorimetric signals. However, some NMNPs also possess powerful enzyme-like catalytic properties, including peroxidase-, oxidase-, hydrolase-, esterase-, phosphatase-, and superoxide-like enzymatic activities, which can increase assay sensitivity by orders of magnitude.^57–59^ Assays based on this concept can be quite simple. For example, Guan et al. recently demonstrated that bare AuNPs exhibit higher peroxidase activity versus various ligand-capped AuNPs. Adding metal ions (Ce^3+^, Fe^2+^ and Cr^3+^) further improved catalytic activity, and an assay for phosphates was developed based on the conversion of a colorless substrate (3,3’,5,5’-tetramethylbenzidine, TMB) to a blue colored oxidation product in the presence of H_2_O_2_.^60^ Another recent example of using NMNP catalytic properties came from Draz et al.,^61^ who labeled PtNPs with a monoclonal antibody against the envelope protein of Zika virus (ZIKV) to detect the virus on-chip (Figure 6a), where gas bubble formation in the presence of H_2_O_2_ was detectable by a smartphone. Sensitivity and specificity of 98.97% and 91.89%, respectively, were achieved under different temperature and humidity conditions with a virus concentration of 250 copies/ mL for ZIKV samples and hepatitis B (HBV) and hepatitis C virus (HCV) patient plasma/serum samples.

Our interest in the catalytic properties of NMNPs has allowed us to develop multiplexed protease nanosensors for *in vivo* early detection of both infectious and noncommunicable diseases.^2^ Molecular-like ultrasmall gold nanoclusters (AuNCs) can function as peroxidase-like enzymes to oxidize chromogenic substrates (e.g., TMB) for signal generation and amplification. This is of particular interest, as they exhibit promising renal clearance properties. Based on our previously reported protease responsive NMNP systems consisting of bioactive peptides,^30,31,33,34,49^ we developed a system whereby catalytic AuNCs (2 nm) were attached to a neutravidin protein core via biotinylated protease-cleavable peptide linkers (AuNC-Nav ~ 11 nm) sensitive to either the zinc-dependent matrix metalloproteinase 9 (MMP9) or the serine protease thrombin, which have been used as biomarkers for cancer and cardiovascular diseases, respectively. Once injected intravenously, the AuNC-NAv dissassembled specifically only when exposed to the relevant dysregulated protease generated at the site of disease. The protease cleavage caused the release of the AuNCs from the core structure into the bloodstream, which were then cleared through the kidneys. The AuNCs were then detected in urine samples through their catalytic activity to oxidise a chromogenic substrate (TMB) in the presence of hydrogen peroxide to a blue-colored solution, easily observable by naked eye (Figure 6b). Compared to healthy mice, the urinary signal increased by 13-fold as measured by the colorimetric readout.

## 4 Toward Infectious Disease Diagnostics at The Point-of-Care Using Noble Metal Nanoparticles

Our development of NP-based biosensors has yielded assays for a variety of noninfectious disease targets over the years, including cancer,^30^ pancreatitis,^42,62^ arthritis,^56^ blood coagulation,^63^ prostate-related diseases,^33^ and cardiovascular disease.^2^ During this time, our attention began to turn to the threat of infectious diseases, as it was apparent that issues such as the increasing prevalence of antimicrobial resistance and the risk of zoonotic or mutant-driven pandemics were not being matched by an increase in invention and translation of viable and field-deployable IVD devices. In 2013, we became founding partners of an Interdisciplinary Research Collaboration (IRC) in the UK, called *i-sense*, funded by the Engineering and Physical Sciences Research Council (EPSRC) to develop digital health systems to test, track, and treat infectious diseases.^64^ Subsequently, we have developed assays targeting various infectious diseases, including influenza,^65—67^ HIV,^3,3,68^ tuberculosis,^69^ and Ebola.^4^

**Figure 7:**
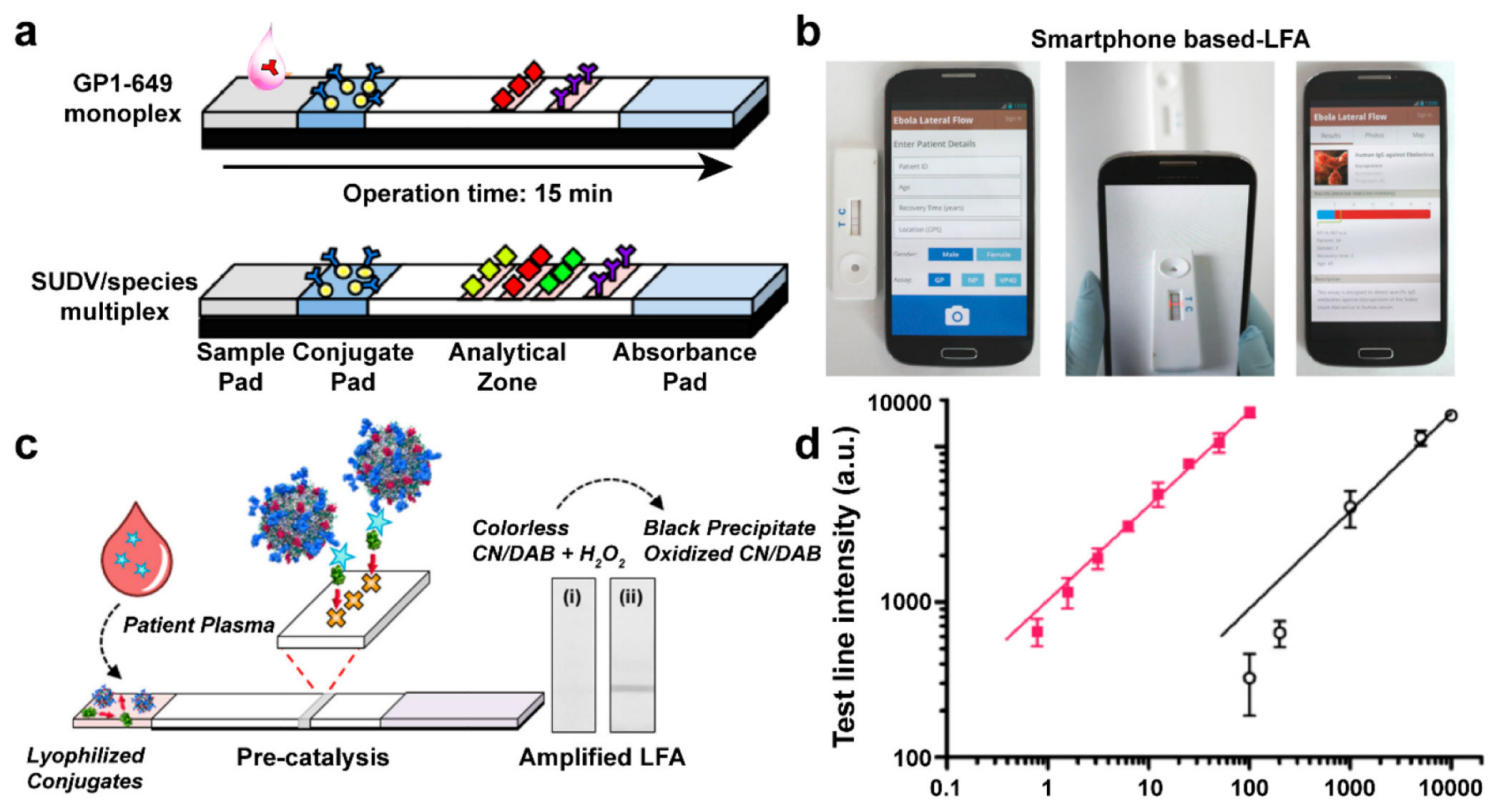
(a) Lateral flow test for Ebola virus IgG detection (diamonds symbolize recombinant proteins, yellow spheres AuNPs, and the Ys antibodies). (b) Integration of a smartphone application for recording patient details. Reproduced from ref 4. Copyright 2018 American Chemical Society. (c) Schematic illustration of catalytic NMNP amplified LFA. (d) Test line intensity illustrating the broad linear dynamic range in spiked sera across 4 orders of magnitude (from <1 pg mL^—1^ to 10^4^ pg mL^—1^) of the protein target before and after amplification. R^2^ = 0.9813. Reproduced from ref 3. Copyright 2018 American Chemical Society.

In the context of infectious disease, actionable information obtained at or near the location of the patient, be it at home, in the field, or in a hospital bed, is the key to better management, early containment and treatment, and improved patient outcomes.^70^ The current state-of-the-art technology is LFAs, which have become a hallmark in many national testing strategies during the COVID-19 pandemic. Particularly, the strong visible color changes due to the SPR variations of NMNPs triggered by analytes offer an elegant solution to enhance the diagnostic readout. Our group developed an LFA POC test comprising anti-human IgG antibody-capped AuNPs (40 nm) and a mobile phone for semiquantitative multiplexed antibody detection.^4^ The designed test strips included a nitrocellulose membrane printed with recombinant viral proteins on the test line and anti-human IgG antibody-capped AuNPs (40 nm) dried on the conjugate pad (Figure 7a), allowing detection of the target antibodies in a sandwich format. We used recombinant viral proteins to detect IgG antibodies against several viral subtypes, including Ebola, Bundibugyo, and Sudan virus, in human serum and validated the technology in Uganda. The LOD of the platform is 200 ng mL^−1^ with an assay time of 15 min for 15 μL of serum. Combined with app-based analysis, semiquantitative results could be achieved by capturing images and analyzing color intensities of the test strip (Figure 7b). With geographic tagging and secure data storage and management, our platform showed great potential as a POC technology to manage recovered patients and improve epidemic control.

The ability of NMNPs to oxidize chromogenic substrates provides a powerful method to improve LFA sensitivity. Inspired by this, we incorporated NMNPs into an LFA test for HIV, targeting p24, which allows disease diagnosis within 2 weeks of infection.^3^ A monoclonal antibody (IgG antibody) was physisorbed onto platinum nanocatalysts (PtNCs) to target p24. An orthogonally biotinylated anti-p24 nanobody was used as the second affinity reagent in the pair. In the presence of p24, the biotinylated nanobody was able to anchor the p24 linked PtNCs to a streptavidin test line on a nitrocellulose membrane (Figure 7c). Nanobodies can be easily produced by bacteria and show high site selectivity to the target epitope. A clear black line could be observed by the naked eye or smartphone imaging when the concentration of analytes ranged from 100 to 10000 pg mL^−1^. PtNCs captured on the test line disproportionated H_2_O_2_ to oxidize a chromogenic substrate (e.g., TMB), generating an unambiguous colored product. Consequently, lower concentrations (1–100 pg mL^−1^) could be detected (Figure 7d). The dual range diagnostic regime empowers smartphones or other image analysis systems to quantitatively or semiquantitatively evaluate the antigen level. Human seroconversion plasma samples from ZeptoMetrix were used to further demonstrate this high sensitivity extending down to low levels of p24 (10.8 pg mL^−1^). The LOD achievable covers the concentration window from early infection to seroconversion (a period over which antibodies develop), which is important for early stage detection.

## 5 Summary and Outlook

Although much research on NMNPs has been conducted, there is still much to learn and much potential to be unlocked, particularly concerning improving signal transduction and protection against nonspecific binding and aggregation. Surface modification of NMNPs with molecules including protein, nucleic acid, drugs, polymers, and peptides is of significant importance to solve these problems and is widely demonstrated for *in vitro* and *in vivo* applications.^71^ Other nanomaterials will be useful for ultrahigh sensitivity including fluorescent materials (quantum dots, upconverting nanoparticles, fluorescent dyes), photothermal or photoacoustic AuNPs, magnetic NPs, electrochemical labels, etc.^72^ Deployment in the field necessitates minimal manual operation. A trade-off must be weighed up, given that the usage of those materials will complicate testing. To ensure the performance of LFAs, pads (detection, conjugate, absorbent), buffers (lysis and immobilization), membranes, and bioreceptors require optimization to achieve sufficient shelf life, further necessitating research into stabilizers like sugars, agars, and gelatins.^73^ Barcode-style assays with each line representing different targets could prove to be the solution toward challenges with multiplexed detection, which is highly desirable for POC testing. The increasing computational power of smartphones facilitates data acquisition, analysis, and processing, which will revolutionize the use of field-deployable IVDs.^1,74,75^

Surface-enhanced Raman spectroscopy (SERS) has proven LOD down to the single-molecule level by taking advantage of electromagnetic properties of confined NMNP surfaces to amplify light scattering up to 10^13^-fold compared to conventional Raman spectroscopy.^76^ Our group has previously exploited SERS and confocal Raman spectroscopy to interrogate and visualize 3D biomolecule distributions in disease progression of infections such as tuberculosis in 3D cell cultures and zebrafish.^77–79^ While reaping the benefits of SERS might be considered a straightforward idea for POC diagnostic, the introduction and manufacturing of nanostructures, surface modifications, and reader instrumentation significantly complicate SERS-based diagnostics. The need for instrumentation increasingly requires our careful reconsideration of not just biosensors and diagnostics but the entire POC testing continuum as well, from research through translation, production, and ultimately reading. Increased focus will be put on the challenge of condensing the physical footprint of test and reader devices, driven by test specifications and rational device design choices, in an effort to make the ultrasensitive NMNP-based tests more economically viable and widely distributable. While a failure to do so is less likely to limit the value of engineering novel biosensor technologies or their applications in established laboratory environments, it does hinder their clinical and commercial translation.

In research, there can sometimes be a polarization of opinion about the relative importance of applied research versus fundamental discovery. In our opinion, both of these avenues are not only important, but indeed crucial which is why we have chosen to make advances in fundamental discoveries and mechanistic understanding as well as always keeping a close eye on what developments are needed to ensure our lab-developed innovations can reach clinical relevance where appropriate. We take great inspiration from how the discovery of the CRISPR-Cas enzyme family, which was born out of observations of anomalies in genetic code^80^, has gone on to revolutionize various areas of application-oriented research, including many high profile examples in IVDs.^81^

The COVID-19 pandemic has brought to attention the drastic need for infectious disease management and pandemic preparedness, and the vital importance of effective IVD devices in these processes. We must build on the hard lessons learned in managing COVID-19 to get ready for other emerging and potentially hard-hitting infectious disease threats. Further, we need to be prepared for the imminent threat of antimicrobial resistance-associated infectious diseases. These are not currently an everyday problem for most people, but should not be underestimated.

We see plenty of opportunities to apply versatile NMNP-based diagnostic technologies integrated with LFAs, SERS, and smartphones, and we are excited to see how the exceptional proliferation of IVDs becomes a widely accessible reality in the coming years. NMNPs-based diagnostic systems and their integration with self-testing devices are increasingly likely to generate breakthroughs to improve health care systems by driving more sensitive detection and more efficient care that overcome the current limitations of detection and enhance the rapid response to both established and emergent infectious diseases.
